# The hypertensive effect of sorafenib is abolished by sildenafil

**DOI:** 10.1186/s40959-020-00064-w

**Published:** 2020-07-13

**Authors:** Hubert Dabiré, Fatou Dramé, Nelly Cita, Bijan Ghaleh

**Affiliations:** 1 U955 - IMRB, Inserm, UPEC, École Nationale Vétérinaire d’Alfort, Créteil, France; 2 INSERM U955 Équipe 03, Faculté de Médecine, 8 rue du Général Sarrail, 94000 Créteil, France

**Keywords:** Sorafenib, Tyrosine kinase inhibitor, Vasorelaxation, Phosphodiesterase-5 (PDE-5) inhibitor, Arterial blood pressure

## Abstract

**Background:**

Contrasting to the well documented tyrosine kinase inhibitor (TKI)-induced hypertension, little is known on their intrinsic vasomotor effects. We investigated the vasomotor effects of sorafenib, a widely used multikinase inhibitor in the treatment of hepatocellular and renal cell carcinoma and tested the hypothesis that sildenafil, a phosphodiesterase-5 (PDE-5) inhibitor, could represent a pharmacological strategy for the treatment of TKI-induced hypertension.

**Methods:**

Concentration-response curves of sorafenib were constructed in endothelium-intact or denuded precontracted rat aorta, in the presence or absence of several inhibitors. Acute intravenous effects of sorafenib on arterial blood pressure were also investigated in anaesthetized rats. Finally, rats were chronically treated with sorafenib during 4 weeks in the presence and absence of sildenafil.

**Results:**

In endothelium intact aortic ring, sorafenib induced a potent concentration-dependent relaxation of precontracted rat aorta. Removal of the endothelium shifted the concentration-response curve of sorafenib to the right and significantly reduced its maximal effects, demonstrating that sorafenib-induced vasorelaxation is endothelium-dependent and endothelium-independent. Inhibition of the different pathways implicated in the endothelium-dependent and independent vasorelaxation revealed that the endothelium-dependent effects of sorafenib result mainly from the activation of prostaglandin and the nitric oxide (NO) pathways. The endothelium-independent vasodilatory effects of sorafenib may result mainly from the activation of Na/K-ATPase and soluble guanylate cyclase. These vasodilatory effects observed in vitro were confirmed by the decrease in arterial blood pressure observed during acute administrations of sorafenib in anesthetized rats. Finally, and most importantly, we report here for the first time that chronic administration of sorafenib in rats induced an increase in SBP that was abolished by sildenafil.

**Conclusion:**

The multikinase inhibitor sorafenib induced in vitro vasorelaxation of large conductance artery, primary by activating soluble guanylate cyclase. Its chronic administration led to arterial blood hypertension that was counteracted by a PDE-5 inhibitor, sildenafil. Our results suggest that targeting the cGMP pathway including NO signalling might be an interesting pharmacological strategy for the treatment of TKI-induced hypertension.

## Background

Tyrosine kinase inhibitors (TKIs) that inhibit growth factors are widely used as targeted therapy for cancers [1–4]. One of their major current side effects is arterial hypertension [5–9]. This increase in blood pressure is considered as a landmark of their anticancer efficacy [5, 9–11]. Different mechanisms have been evoked such as direct inhibition of nitric oxide (NO) production, increased endothelin-1 release, NO deficiency-mediated increased proliferation of vascular medial cells, rarefaction of small arteries and arterioles or increased arterial stiffness [5, 9, 12–15]. Regarding the clinical management of this hypertension, current agents available for the treatment of essential hypertension are the same used for the treatment of TKIs-induced hypertension [6, 9, 16, 17].

This hypertensive effect of TKIs has also been observed in rodent and large animal models [18–23] but in vitro investigations on vessel rings are not always consistent with these observations. Imatinib, nilotinib and sorafenib induced concentration-dependent relaxation of rat pulmonary artery [24] and sunitinib blunted endothelin-1-induced vasoconstriction, reduced phenylephrine-induced maximal vasoconstriction and facilitated acetylcholine (Ach) -induced vasodilation of rat renal resistance arteries [22], showing a vasodilatory property of sunitinib on renal resistance arteries. This suggests that the in vivo effects of TKIs might not be the consequences of direct vasomotor properties. However, sunitinib produced vasoconstriction selectively in the systemic vascular bed without affecting pulmonary or coronary circulations in swine [20]. The reasons for these discrepancies remain unknown.

Accordingly, we decided to decipher the vasomotor properties of sorafenib which is widely used for the treatment of hepatocellular and renal cell carcinoma [1, 25, 26]. Sorafenib is a multikinase inhibitor, acting preferentially on vascular endothelial growth factor receptor (VEGFR), platelet derived growth factor receptor (PDGFR) and c-RAF (not a tyrosine kinase receptor). Our second goal was to investigate the in vivo effects of acute and chronic administration of sorafenib in rats. As our in vitro results showed major intervention of the cGMP-pathway in the vasomotor effects of sorafenib, we next hypothesized that phosphodiesterase-5 (PDE-5) inhibition with sildenafil [27–29] might represent an interesting strategy to counteract sorafenib-induced increase in arterial blood pressure. PDE-5 is cGMP selective and is expressed in numerous organs, e.g. smooth muscle, heart and kidney [30, 31]. By preventing the degradation of cGMP to 5’GMP, inhibition of PDE-5 increases NO availability leading to potentiation of the NO-dependent vasodilatory effects.

## Methods

### Animals

Twelve-week old male Wistar rats (Janvier Labs, Le Genest-St-Isle, France) were maintained under 12 h light-dark cycle with free access to food and water. They were allowed a week period of acclimation in our laboratory animal facilities. All the experiments were carried out in accordance with current institutional guidelines for the care and use of experimental animals. They were approved by our local animal ethical committee [ComEth AFSSA-ENVA-UPEC approval #12/09/17–3].

### Aortic rings preparation

In vitro experiments were performed in isolated aortic rings as previously described [32–34]. Under pentobarbital anaesthesia (60 mg/kg ip), the thoracic aorta was carefully excised and placed in cold Krebs solution containing (mM): 118.3 NaCl, 4.7 KCl, 2.5 CaCl_2_, 1.2 MgSO_4_, 1.2 KH_2_PO_4_, 25 NaHCO_3_, 0.016 EDTA and 11.1 glucose. The aorta was cleaned of excess connective tissue and fat and cut into rings of approximately 3–4 mm in length. Special care was taken to avoid damaging the luminal surface of the endothelium. In experiments on endothelium-denuded rings, the endothelium was removed by gently rubbing the intimal surface with the tip of small forceps. Aortic rings were suspended in 10 ml organ baths filled with Krebs solution continuously aerated with a mixture of 5% CO_2_, 95% O_2_, pH 7.4, at 37.4 °C. One end of the aortic ring was connected to a tissue holder and the other to an isometric force transducer (EMKABath4, EMKA Technologies, Paris, France). Rings were progressively stretched to a resting tension of 2 g during 120 min. During this equilibration period, the rings were washed every 20 min. Then, a first relaxation to acetylcholine (Ach, 10^− 4^ M) was implemented to check the integrity or the absence of the endothelium in rings precontracted with noradrenaline (NA, 3.10^− 8^ M). Rings were equilibrated again to baseline tension during 90 min by rinsing with Krebs solution.

### In vitro effects of sorafenib and sildenafil

We examined the concentration-response curves of sorafenib (10^− 10^ - 10^− 4^ M) in a first series of experiments. We also compared them to those of Ach (10^− 10^ - 10^− 4^ M) and sodium nitroprusside (SNP, 10^− 10^ - 10^− 4^ M) in endothelium-intact and endothelium-denuded aortic rings. For this purpose, the rings were incubated during 20 min with the solvent of each agent (dimethyl sulfoxide, DMSO, 90 μl for sorafenib or distilled water, 90 μl for Ach and SNP). Then after, cumulative concentration-response curves to sorafenib, Ach and SNP were constructed after precontraction with NA (3.10^− 6^ M). Each concentration of the drugs was added at the maximal effect of the precedent concentration. Each ring was subjected to one concentration-response curve. The concentration-response curves were continuously recorded on a personal computer by means of IOX software v 2.9.4.35 (EMKA Technologies, Paris, France) for further analysis (Datanalyst software v 2.6.1.13, EMKA Technologies, Paris, France). In addition, the effects of sildenafil (10^− 10^ - 10^− 4^ M) on intact rat aortic rings were investigated in similar conditions and compared to those of Ach and SNP.

In a second series of experiments, the mechanisms of the vasorelaxation induced by sorafenib were investigated in endothelium-intact and endothelium-denuded aortic rings. The rings were incubated during 20 min with different inhibitors (Table 1) or their combinations. Then after, cumulative concentration-response curves to sorafenib (10^− 10^ - 10^− 4^ M) were constructed as described above. Each ring was subjected to one inhibitor and one concentration-response curve.

**Table 1:** Inhibitors of the different pathways implicated in vasorelaxation

Drugs	Selectivity	Concentration (M)	References
U73122	Phospholipase C	10^−5^	[35]
Manoalide	Phospholipase A_2_	3.10^−7^	[36]
L-NAME	Nitric oxide (NO) synthase	3.10^− 5^	[37]
Indomethacin (INDO)	Cyclo-oxygenase (COX)	3.10^− 5^	[38, 39]
Rofecoxib	Prostacyclin (PGI_2_) synthase	3.10^−7^	[38–40]
ODQ	Soluble guanylyl cyclase (sGC)	10^−5^	[41–43]
Ouabain	Na/K-ATPase	10^−4^	[44]

U73122, 1-[6-{[17β-3-methoxyestra-1,2,3(10)-trien-17-yl]amino}hexyl]-1H-pyrrole-2,5-dione; L-NAME, N^ω^-Nitro-L-arginine methyl ester; ODQ, 1H -[1, 2, 4] Oxadiazolo [4,3-a]quinoxalin-1-one

The quantification of the results was performed by calculating the maximal effect (E_max_), the concentration inducing 50% of E_max_ (EC_50_) and pEC_50_ (−log (EC_50_)).

### Acute effects of sorafenib on blood pressure in anaesthetized rats

Arterial blood pressure (BP) was recorded as previously described [45]. Briefly, under anaesthesia with pentobarbital sodium (60 mg/kg ip), a polyethylene catheter [a PE-10 (0.28 mm ID, 0.61 mm OD; Clay Adams, Parsippany, NJ) fused to a PE-50 (0.58 mm ID, 0.96 mm OD; Guerbet, Louvres, France)] filled with heparinized 0.9% NaCl (50 U/ml) was inserted into the common carotid artery and connected to a signal processor (MP35, Biopac Systems Inc., CA, USA) via a pressure transducer (BP-T, EMKA Technologies, Paris, France). Blood pressure signals were recorded online at a sampling rate of 250 points/s (BSL Pro 3.7, Biopac Systems Inc., CA, USA). Another catheter was introduced into the femoral vein for drugs administration. After 30 min of stabilisation, increasing doses of sorafenib were administered intravenously as a bolus (0.1, 0.3 and 1 mg/kg). Each dose was given after blood pressure had returned to baseline value and each rat was subjected to one dose-response curve.

### Chronic effects of sorafenib on blood pressure in conscious rats

Non-invasive tail-cuff measurement of systolic blood pressure (SBP) was used to follow the changes in blood pressure during the four-weeks treatment protocol [46]. The rats were kept in a rodent restrainer in a quiet room. The tail was placed on a piezoelectric sensor connected to a Powerlab 2/20 (ADInstruments Pty Ltd., Australia) and, around an inflator, connected to a NIBP Controller (ADInstruments Pty Ltd., Australia). The NIBP Controller was connected to the Powerlab 2/20 which itself was connected to a personal computer allowing recording of pressure waveforms by mean of Chart® software (ADInstruments Pty Ltd., Australia).

Rats were trained once a day during 5 days before basal SBP recording. They were then randomly assigned to 5 groups: a control, untreated group (CTRL), a DMSO-treated group (DMSO, 1 ml/kg/d), a sorafenib-treated group (SORA, 30 mg/kg/d), a sildenafil-treated group (SILD, 3 mg/kg/d) and a group treated by sorafenib plus sildenafil (SOSI, 30 mg/kg/d plus 3 mg/kg/d, respectively). SBP was recorded in the morning, every day during the 5 first days of the treatment and every week then after. Drugs were given by gavage. At the end of the 4-weeks treatments, the rats were anesthetized, and aorta and end organs were harvested for in vitro investigations.

### Drugs

Acetylcholine (Ach), sodium nitroprusside (SNP), L-NAME, Ouabain and noradrenaline (NA) (Sigma Aldrich, St Quentin Fallavier, France) were dissolved in distilled water. Indomethacin and U73122 (Sigma Aldrich, St Quentin Fallavier, France), sorafenib, ODQ, rofecoxib, sildenafil and manoalide (CliniSciences, Nanterre, France) were dissolved in DMSO. The selectivity and the doses are indicated in Table 1.

### Statistical analysis

Values are means ± SEM of the number of rats. Statistical analysis was performed with StatView software (v5, Abacus Concepts Inc.). ANOVA for repeated measures and one factor ANOVA followed by Fisher Protected Least significance were used to compare the effects of drugs. Differences were considered significant at *P* < 0.05.

## Results

### Vasomotor effects of sorafenib

**Fig. 1:**
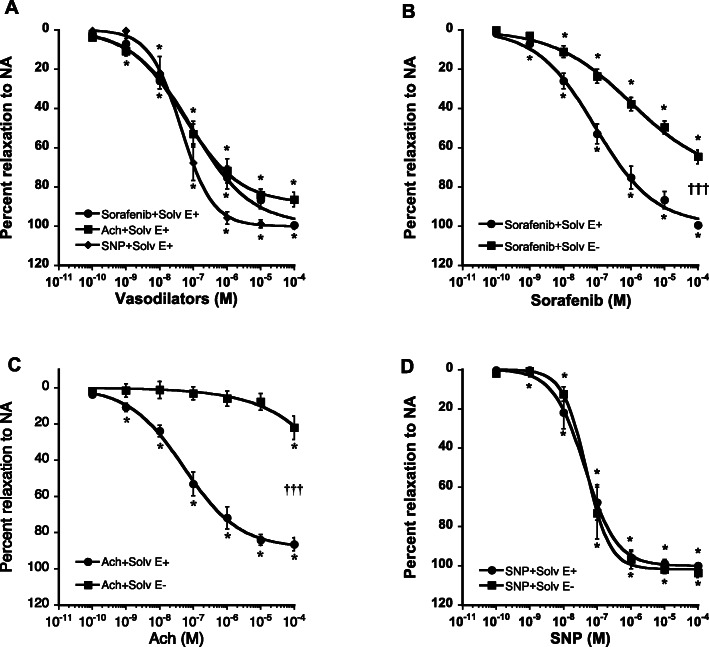
Comparative concentration-response curves (**a**) of sorafenib (full circles), acetylcholine (Ach, full squares), and sodium nitroprusside (SNP, full diamonds) in endothelium intact rat aortic rings precontracted by NA (3.10^− 6^ M). Concentration-response curves of sorafenib (**b**), Ach (**c**) and SNP (**d**) in endothelium intact (full circles) and denuded (full squares) rat aortic rings precontracted by NA (3.10^− 6^ M). Each point is the mean ± SEM of 6–15 rats. * *P* < 0.001 vs 10^− 10^ M; ††† *P* < 0.001 vs endothelium intact ring. ANOVA for repeated measures

In endothelium-intact aortic rings, sorafenib exhibited concentration-dependent vasorelaxation similar to the effects of Ach and SNP (Fig. 1a). Removal of the endothelium almost abolished the vasorelaxation induced by Ach (E_max_ = 86 ± 4 vs 22 ± 7; *P* < 0.001) and did not change that of SNP (E_max_ = 100 ± 1 vs 104 ± 2), confirming the absence of functional endothelium and preserved vascular smooth muscle function (Fig. 1c and d). Endothelium removal shifted the concentration-response curve of sorafenib to the right and reduced its E_max_ from 99 ± 1 to 65 ± 4% (Fig. 1b). These experiments revealed that sorafenib induces vasorelaxation through both endothelium-dependent and -independent mechanisms.

**Fig. 2:**
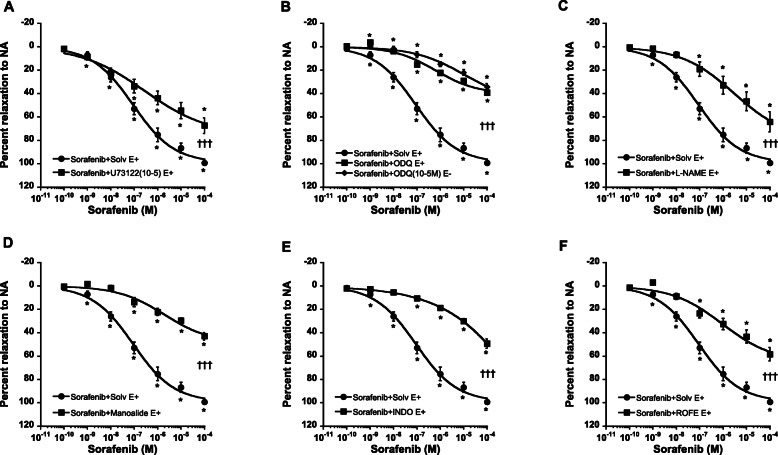
Concentration response curves of sorafenib in endothelium intact rat aortic rings, in the presence of solvent (Solv, full circles) or different inhibitors (full squares): U73122 (**a**), L-NAME (**b**), ODQ (**c**; full diamonds, endothelium denuded), manoalide (**d**), indomethacin (INDO, **e**) and rofecoxib (ROFE, **f**). Each point is the mean ± SEM of 5–15 rats. * *P* < 0.001 vs 10^− 10^ M; ††† *P* < 0.001 vs Solvent. ANOVA for repeated measures

Vasorelaxation may result from direct effect on vascular smooth muscles or the release of a multiple endothelium-derived relaxing factors [47, 48] particularly NO and prostacyclin (PGI_2_). In endothelium intact aortic rings, inhibition of phospholipase C (PLC) by U73122 (10^− 5^ M; Fig. 2a) or NO synthase by L-NAME (3.10^− 5^ M; Fig. 2b) shifted the concentration-response curves of sorafenib to the right and significantly reduced its maximal effects (E_max_ = 67 ± 7% and 64 ± 9% vs 99 ± 1%). Inhibition of soluble guanylyl cyclase (sGC) by ODQ (10^− 5^ M; Fig. 2c) in the presence or the absence of the endothelium, produced similar effects.

Regarding the PGI_2_ pathway, inhibition of phospholipase A_2_ (PLA_2_) by manoalide (3.10^− 7^ M; Fig. 2d), COX by indomethacin (INDO, 3.10^− 5^ M; Fig. 2e) or PGI_2_ synthase by rofecoxib (3.10^− 7^ M; Fig. 2f) shifted the concentration-response curve of sorafenib to the right and significantly reduced its maximal effects in endothelium intact aortic rings (E_max_ = 43 ± 3%, 49 ± 4% and 58 ± 6% vs 99 ± 1%, respectively).

**Fig. 3:**
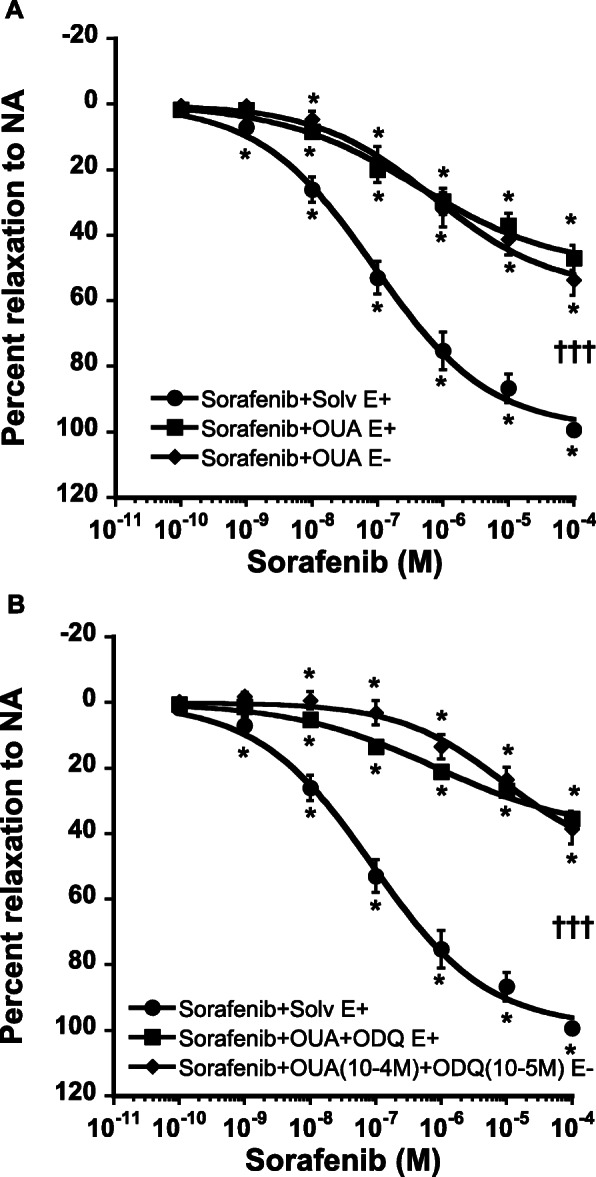
Concentration response curves of sorafenib in endothelium intact (E+) and endothelium denuded (E-) rat aortic rings, in the presence of solvent (Solv, full circles), ouabain (OUA, full diamonds, Panel **a**) or the combination of OUA plus ODQ (full diamonds, Panel **b**). Each point is the mean ± SEM of 7–8 rats. * *P* < 0.001 vs 10^− 10^ M; ††† *P* < 0.001 vs Solvent. ANOVA for repeated measures

In endothelium intact rings, inhibition of Na/K-ATPase by ouabain (OUA, 10^− 4^ M) significantly reduced sorafenib-induced vasorelaxation and this effect was not altered by endothelium removal (Fig. 3a), E_max_ values being similar (47 ± 4% vs 54 ± 5%). In addition, endothelium removal did not alter the effects of combined inhibition of soluble guanylyl cyclase by ODQ and Na/K-ATPase (Fig. 3b).

### Vasomotor effects of sildenafil

**Fig. 4:**
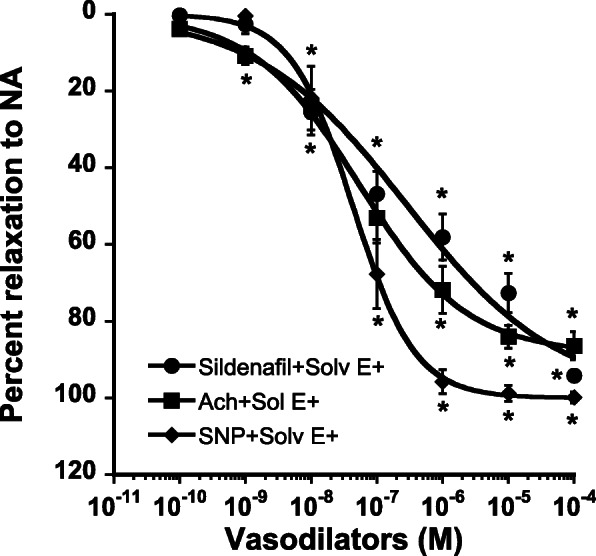
Comparative concentration-response curves of sildenafil (full circles), acetylcholine (Ach, full squares), and sodium nitroprusside (SNP, full diamonds) in endothelium intact rat aortic rings precontracted by NA (3.10^− 6^ M). Each point is the mean ± SEM of 7–9 rats. * *P* < 0.001 vs 10^− 10^ M. ANOVA for repeated measures

In endothelium-intact aortic rings, sildenafil exhibited concentration-dependent vasorelaxation similar to the effects of Ach and SNP (Fig. 4) with comparable maximal effect (94 ± 1% vs 86 ± 4% and 100 ± 1% for Ach and SNP, respectively).

### In vivo effects of acute administration of sorafenib in normotensive rats

**Fig. 5:**
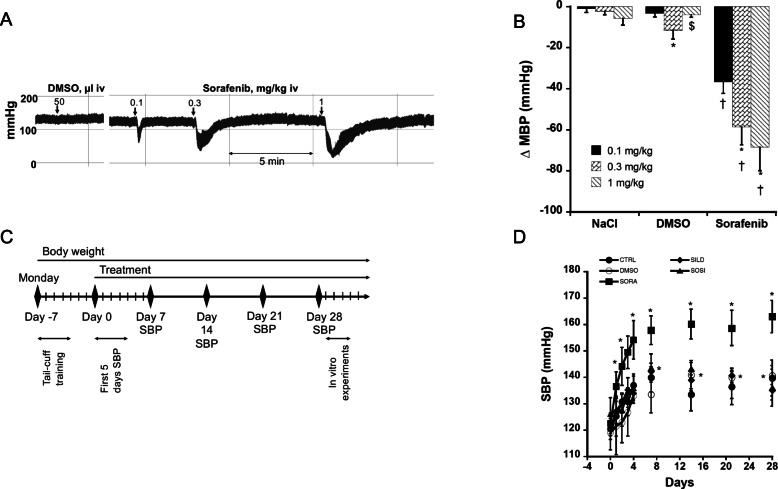
In vivo effects of sorafenib. Panel **a**: trace recording of blood pressure in anaesthetised rats receiving acute administration of dimethyl sulfoxide (DMSO, 50 μl at 1/1000) or sorafenib. Panel **b**: Dose-response curve of sorafenib (0.1, 0.3 and 1 mg/kg iv) on mean blood pressure (MBP). Each bar is the mean ± SEM of 6 rats. * *P* < 0.05; ** *P* < 0.01 compared to the first dose; †† *P* < 0.01, ††† *P* < 0.001 compared to NaCl and DMSO. One factor ANOVA followed by Fisher PLSD. Panel **c**: protocol for chronic treatments. Panel **d**: Evolution of systolic blood pressure (SBP) in conscious normotensive rat for 4 weeks treatment with sorafenib. Each point is the mean ± SEM of 4–6 rats. * *P* < 0.05 compared to Day 0; † *P* < 0.05 compared to Control

The effects of acute administration of sorafenib were investigated in normotensive anesthetized rats. Intravenous administration of the solvent (DMSO) did not change arterial blood pressure. In contrast, intravenous bolus administration of sorafenib (0.1, 0.3 and 3 mg/kg) induced a dose-dependent decrease in arterial blood pressure (Figs. 5a and b). This effect was short-lasting and arterial blood pressure rapidly returned to baseline values as illustrated in Fig. 5a.

### In vivo effects of chronic sorafenib administration and its combination with sildenafil

Animals received chronic treatments (DMSO, SORA, SILD, SOSI) according to the protocol illustrated in Fig. 5c. No difference in SBP was observed among the 5 groups of rats before starting treatments (121 ± 4 mmHg, 119 ± 6 mmHg, 123 ± 4 mmHg, 121 ± 3 mmHg and 126 ± 6 mmHg in control, DMSO, SORA, SILD and SOSI groups, respectively). As illustrated in Fig. 5d, after an initial increase in SBP at the beginning of the protocol, SBP remained stable throughout the protocol in control animals. DMSO-treated animals showed similar SBP values than controls. In contrast, chronic administration of sorafenib rapidly induced a significant and stable increase in SBP. Interestingly, sildenafil did not significantly change SBP compared to control and DMSO groups but abolished the hypertensive effect of sorafenib with normalised values of SBP (Fig. 5d).

**Fig. 6:**
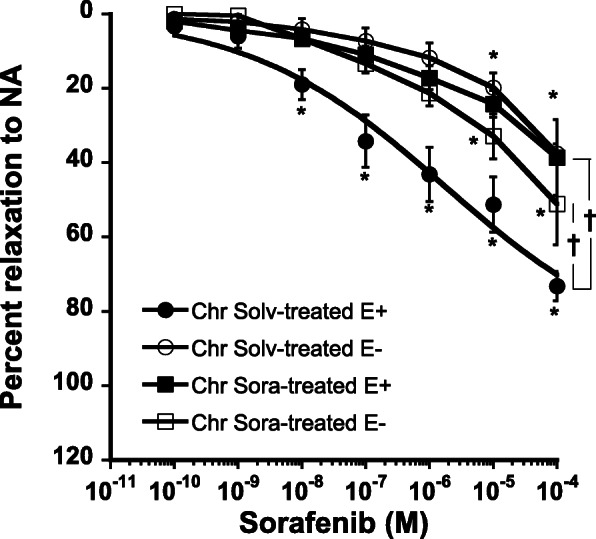
Concentration response curves of sorafenib in endothelium intact (E+) or denuded (E-) aortic ring of rats treated for 4 weeks with solvent (chr Solv) or sorafenib (chr Sora). Mean ± SEM of 3–6 rats. * *P* < 0.01 vs first concentration. † *P* < 0.05 comparison of the different curves. Anova for repeated measures

Chronic oral administration of sorafenib altered endothelium-dependent vasodilatory properties of sorafenib. We observed significantly reduced vasorelaxation when sorafenib was added to the aortic rings as compared to aortic rings obtained from vehicle treated animals (E_max_ = 39 ± 10% vs 73 ± 4%; *P* < 0.001) and more importantly, endothelium removal did not alter this response (Fig. 6).

## Discussion

Notwithstanding the well documented tyrosine kinase inhibitor (TKI)-induced hypertension, little is known surprisingly on their intrinsic vasomotor effects. We investigated the vasomotor effects of sorafenib, a widely used multikinase inhibitor in the treatment of cancers and tested the hypothesis that sildenafil, a phosphodiesterase-5 inhibitor, could represent a pharmacological strategy for the treatment of TKI-induced hypertension. In endothelium intact aortic ring, sorafenib induced a potent concentration-dependent relaxation of precontracted rat aorta. Removal of the endothelium shifted the concentration-response curve of sorafenib to the right and significantly reduced its maximal effects, demonstrating that sorafenib-induced vasorelaxation involves a dual mechanism, i.e., endothelium dependent and endothelium independent vasorelaxation. To determine the pharmacological mechanisms implicated in the sorafenib-induced vasorelaxation, we investigated the effects of several inhibitors. The results obtained revealed that the endothelium dependent effects of sorafenib result mainly from the activation of prostaglandin and the nitric oxide pathways. The endothelium independent vasodilatory effects of sorafenib may result mainly from the activation of Na/K-ATPase and soluble guanylate cyclase. These vasodilatory effects observed in vitro were confirmed by the decreases in arterial blood pressure observed during acute administrations of sorafenib in anesthetized rats. Finally, and most importantly, we report here for the first time that sildenafil abolished the increase in systolic blood pressure induced by chronic administration of sorafenib in rats.

Hypertension is one of the most frequently reported adverse effects associated with angiogenesis inhibitors, including sorafenib. Sorafenib-induced hypertension can occur in patients as early as within the first day from initiating therapy and is easily detectable after 1 week of treatment [49–52]. Similar results have been reported with sorafenib and sunitinib given by gavage in Wistar rats and Sprague-Dawley rats, regardless of whether blood pressure was recorded by telemetry or tail-cuff. The changes in arterial blood pressure were dose-dependent and appeared in the first week after starting the treatment [18, 21, 23]. In the present study, at the end of the treatment, SBP reached normotensive values of 140 mmHg in CTRL, DMSO and SILD groups. Sorafenib induced hypertension with increased SBP to 160 mmHg. The onset of sorafenib-induced increase in SBP was similar to that already reported. In vitro investigations showed that the vasorelaxation induced by sorafenib was almost abolished in aortic rings obtained from chronically sorafenib-treated rats. In this context, in conscious rats, chronic administration of sildenafil normalized values of systolic blood pressure from the very first days of sorafenib treatment and overall, throughout the protocol, sildenafil abolished the hypertensive effect of sorafenib and SBP values were similar to those of CTRL and DMSO groups. In our experimental conditions, sildenafil per se induced vasorelaxation as previously reported and ascribed to amplification of NO/cGMP pathways through inhibition of PDE-5 [28, 53]. This suggests that acting on cGMP pathway including NO signalling may represent a suitable strategy for the treatment of sorafenib-induced hypertension.

The present experiments report the effects of sorafenib on isolated large conductance artery, the rat aorta, and the pharmacological mechanisms implicated. In rat aorta precontracted by noradrenaline, sorafenib induced concentration-dependent vasorelaxation, the pharmacological mechanism of which appeared complex. The reduction of the effects of sorafenib after endothelium removal suggests that its vasodilatory effects are both endothelium dependent and endothelium independent. Many mechanisms are implicated in the endothelium dependent and independent vasorelaxation [47, 48]. Our results led us to conclude that sorafenib-induced vasorelaxation involves NO, prostaglandins, cGMP and Na/K-ATPase. However, since combined inhibition of different pathways did not completely suppress the effects of sorafenib, other mechanisms could not be excluded, as hydrogen peroxide or epoxyeicosatrienoic acid pathways, although their importance might be negligible.

Sorafenib-induced vasorelaxation in vitro appeared paradoxical in regard to the increase in blood pressure in vivo. However, direct activation of growth factor receptors as EGFR and PDGFR induced vasoconstriction of aorta from normotensive, spontaneously hypertensive or 1 kidney, 1 clip (1 K,1C) hypertensive rats [54–57]. Thus, it seems not surprising that inhibition of these growth factor receptors by TKIs would induce vascular smooth muscle cell relaxation and vasodilatation as reported in rat pulmonary arteries [24] and aorta (the present results).

It should be acknowledged that the effects of sorafenib on resistance vessels were not assessed in the present study. These vessels are the most implicated in the control of peripheral resistance. However, it is likely that the vasodilatory effects observed in large conductance arteries could also be observed in these small arteries as in the present study, bolus intravenous administrations of sorafenib decreased arterial blood pressure. These results are also in agreement with previous reports showing that TKIs induce vasorelaxation in pulmonary and renal arteries [22, 24]. Finally, we did not directly assess endothelium removal with markers such as CD31. However, in all rings, we ensure the functional presence or absence when endothelium was removed using acetylcholine, a well-recognized approach.

Two other aspects need also to be discussed. First, the long-term use of sildenafil might have impact on penile erection. In our experiments, simple observation of the rats without any specific measurement did not reveal that chronic treatment with sildenafil induced penile erection. One could hypothesize that the doses used in the present study are lower than those required to induce penile erection. However, our study was not designed to allow such conclusion and specific investigations are needed to address this issue. It should be also acknowledged that the rats did not have tumors in the present study. Therefore, further experiments are needed to verify that sildenafil does not interact with the anticancer properties of sorafenib experimentally in a rat model of hepatocellular carcinoma [58] before stuying this strategy in patients.

## Conclusions

The present results show that the multikinase inhibitor sorafenib induced in vitro vasorelaxation of large conductance artery, primary by activating soluble guanylate cyclase. Its chronic administration led to arterial blood hypertension that was counteracted by a PDE-5 inhibitor, sildenafil. Our results suggest that targeting the cGMP pathway including NO signalling might be an interesting pharmacological strategy for the treatment of TKI-induced hypertension.

## Data Availability

Not applicable.

## Abbreviations

Ach: Acetylcholine
BP: Blood pressure
COX: Cyclo-oxygenase
DMSO: Dimethyl sulfoxide
INDO: Indomethacin
NA: Noradrenaline
NO: Nitric oxide
ODQ: (1H-[1,2,4]oxadiazolo[4,3-a]quinoxalin-1-one)
OUA: Ouabain
PDGF: Platelet Derived Growth Factor
PGI_2_: Prostacyclin
SBP: Systolic blood pressure
SILD: Sildenafil
SORA: Sorafenib
SOSI: Sorafenib + sildenafil
TKIs: Tyrosine kinase inhibitors
U73122: (1-[6-{[17β-3-methoxyestra-1,2,3(10)-trien-17-yl]amino}hexyl]-1H-pyrrole-2,5-dione)
VEGF: Vascular endothelial growth factor
